# *Legionella pneumophila*—*Klebsiella pneumoniae* Pulmonary Coinfection in a COVID-19 Patient: Case Report

**DOI:** 10.3390/idr16060085

**Published:** 2024-11-11

**Authors:** Maria Irina Brumboiu, Edina Iuga, Andreea Ivanciuc, Irina Iaru, Alexandru Durla-Pașca, Pavel Șchiopu, Adrian Gabriel Pană

**Affiliations:** 1 Medical Specialties Department, Iuliu Hatieganu University of Medicine and Pharmacy, 400 012 Cluj-Napoca, Romania; iuga.edina@elearn.umfcluj.ro (E.I.); ivanciuca98@gmail.com (A.I.); durla.alex@yahoo.com (A.D.-P.); feathera.40@gmail.com (A.G.P.); 2 Cluj Unit, French-Speaking International Clinical Epidemiology Network, Iuliu Hatieganu University of Medicine and Pharmacy, 400 012 Cluj-Napoca, Romania; 3 Pharmacology, Physiology, Pathophysiology Department, Iuliu Hatieganu University of Medicine and Pharmacy, 400 012 Cluj-Napoca, Romania; 4 Microbiology Department, Iuliu Hatieganu University of Medicine and Pharmacy, 400 012 Cluj-Napoca, Romania; pavel.schiopu@umfcluj.ro

**Keywords:** pneumonia, *Legionella pneumophila*, *Klebsiella pneumoniae*, COVID-19, coinfection, case report

## Abstract

**Background.** Pulmonary superinfections increase the mortality risk among COVID-19 patients, highlighting the need for enhanced understanding to enable early and accurate diagnosis. **Methods.** We present the case of a patient, a 76-year-old man, hospitalized for a severe form of COVID-19, with a ground-glass pneumonia, involving 40–45% of lung surfaces. **Results.** In evolution, the clinical condition worsened, presenting leukocytosis with neutrophilia, imaging towards resorption, and computer tomography images showing the appearance of pulmonary condensations in the right lower lobe, the posterior portion of the left lower lobe and pleural collections. Carbapenemase-producing *Klebsiella pneumoniae* was isolated from the tracheal aspirate, and the real-time polymerase chain reaction test was positive for *Klebsiella pneumoniae* and *Legionella pneumophila*. The investigations that were carried out allowed us to establish the coinfections as a probable case of Legionnaire’s disease and a ventilator-associated pneumonia with *Klebsiella pneumoniae*. **Conclusions.** The case analysis revealed that rare pneumonias may remain undiagnosed, and coinfections may be conditioned by pathophysiological factors or components of COVID-19 critical form treatment. Enhanced understanding of these aspects in clinical practice may contribute to reducing mortality risk in COVID-19 patients.

## 1. Introduction

Pulmonary superinfections worsen the clinical outcomes in COVID-19 patients. The presence of coinfections has been noted since the beginning of the pandemic, involving bacterial, fungal, or viral pathogens [1,2,3]. Their frequency has been found to be high among hospitalized COVID-19 patients, requiring intensive care [4]. In these patients, a meta-analysis conducted for the January–April 2020 period of the pandemic, showed that the risk of death was 5.82 (95% CI: 3.4–9.9) times higher than in those without coinfections [5].

Among the bacterial species of pulmonary superinfections, both *Legionella pneumophila* and *Klebsiella pneumoniae* have been reported and are published in medical article [6]. However, cases involving simultaneous coinfection with these two etiological infective agents in COVID-19 patients have not yet been documented in the literature.

In the case of Legionnaires’ disease, the risk of mortality is known to increase with delays in administering the appropriate antibiotic treatment [7,8]. Additionally, the literature reports instances of Legionnaires’ disease with viral (influenza) or bacterial coinfections (*Mycoplasma pneumoniae*, *Chlamydia* spp., *Streptococcus pneumoniae*, *Klebsiella pneumoniae*, *Pseudomonas aeruginosa*), which exacerbate clinical outcomes, particularly in immunocompromised patients, such as those with organ transplants [9,10].

Legionnaires’ disease is rarely diagnosed in our geographical region, the Cluj county, in Romania, which led to a lack of monitoring for its potential association with COVID-19 cases [11]. However, in France, a retrospective study for the year 2020, highlighted that most cases of coinfection with *Legionella* in COVID-19 patients were reported from the region that usually diagnoses the most cases of legionellosis [12]. These observations, along with the hypercortisolemia that predispose patients to infections and the recent immunomodulatory treatment for COVID-19, support the need for further investigation into coinfections and treatments employed [13,14].

In practice, the recognition of pulmonary coinfections in COVID-19 patients is hampered by the similarity of symptoms, as well as the fact that a severe form of pneumonia can be caused by other infectious agents (such as *Legionella pneumophila*) therefore it become difficult to differentiate whether the severity is due to primary pneumonia or a pulmonary superinfection [7]. Early diagnosis of polymicrobial pulmonary superinfection is crucial for the prompt selection and administration of appropriate treatment, thereby increasing the likelihood of patient survival. In this context, analyzing individual cases of patients with severe COVID-19 may serve as an initial observational step toward outlining potential risk factors contributing to the development of pulmonary coinfections.

We present the case of a patient with a severe form of COVID-19, who during hospitalization developed pulmonary superinfection with *Legionella pneumophila* and *Klebsiella pneumoniae*.

## 2. Case Report

The patient, a 76-year-old male, from an urban environment, was admitted at the beginning of November 2021 to a general hospital for dyspnea, dry cough, fever (39 °C), headache, asthenia, dizziness. These symptoms had started seven days prior and had progressively worsened. His medical history included essential arterial hypertension, atrial fibrillation with moderate ventricular rate, colon cancer diagnosed 6 years prior (surgically treated, chemotherapy, and radiotherapy), and benign prostatic hypertrophy. The patient had been vaccinated against COVID-19 with two doses of the mRNA vaccine, four months earlier (in June). Clinical observations included decreased vesicular breath sounds, disseminated bilateral basal crackles, a blood pressure of 130/80 mmHg, a heart rate of 90 beats per minute, and an arterial blood oxygen saturation (SaO_2_) of 80%. Notably, the patient was overweight with a body mass index of 29.4 kg/m^2^ (height 165 cm, weight 80 kg) and presented a postoperative abdominal scar from colon cancer surgery.

Prior to the onset of the illness, he lived alone, he had not traveled, and interpersonal contacts were limited to visits from two family members and to obtaining necessary supplies and medication from the pharmacy.

The patient was receiving chronic treatment for his medical conditions with beta-blockers (metoprolol 50 mg), a diuretic (indapamide 1.5 mg), ergot alkaloids (nicergoline 30 mg), a combination of HMG CoA reductase inhibitors and calcium channel blockers (atorvastatin with amlodipine 10/5 mg), and an alpha-adrenoreceptor antagonist (tamsulosin hydrochloride 0.4 mg). The patient’s previous colon cancer was in remission and no longer required cancer treatment.

Initially, a mild inflammatory syndrome was observed with C-reactive protein (CRP) at 24 mg/dL and erythrocyte sedimentation rate (ESR) of 30 mm at one hour and 70 mm at two hours (Table 1). Aspartate aminotransferase (AST) was slightly elevated at 55 U/L, and hypokalemia (2.8 mEq/L) was noted. Hypoxemia, measured by arterial partial pressure of oxygen (PaO_2_), ranged between 47 and 82 mmHg during oxygen therapy. Real-time polymerase chain reaction (rt-PCR) for SARS-CoV-2 was positive four days prior hospitalization.

**Figure 1:**
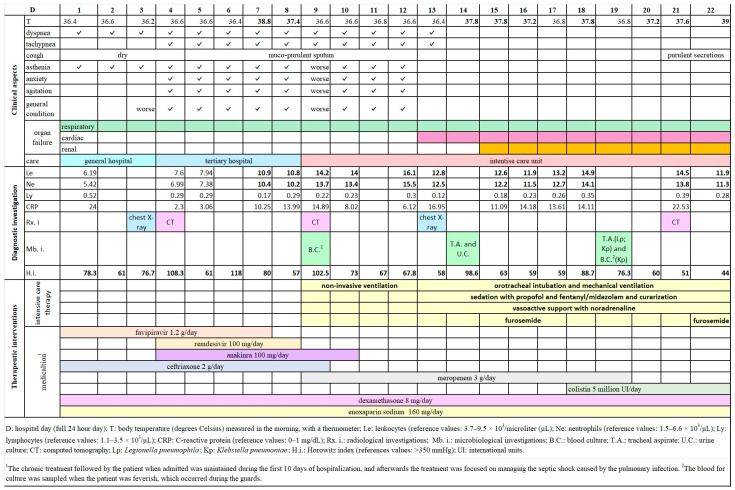
Clinical, diagnostic, and therapeutic aspects, and their evolution during the entire period of the patient’s hospitalization. In the figure: bold numbers for diagnostic investigations indicate pathological values; the symbol “✓” marks the presence of certain specific clinical aspects for each hospitalization day; colored horizontal cells and bars illustrate the duration of certain clinical aspects, of the type of healthcare provided, and the treatment administered.

Hematological parameters showed a sharp increase in leukocytes, with neutrophilia on the seventh day and a subsequent second rise on the 12th day of hospitalization, which persisted thereafter (Figure 1). Transaminases, particularly alanine aminotransferase (ALT), as well as CRP, ferritin, fibrinogen, and urea level were elevated. There was a decrease in red blood cells, hemoglobin, and hematocrit, likely due to occult digestive bleeding detected by the immunochromatographic method in stool samples on the sixth day of hospitalization. Other hematological, biochemical, and immunological parameters were within normal limits.

**Figure 2:**
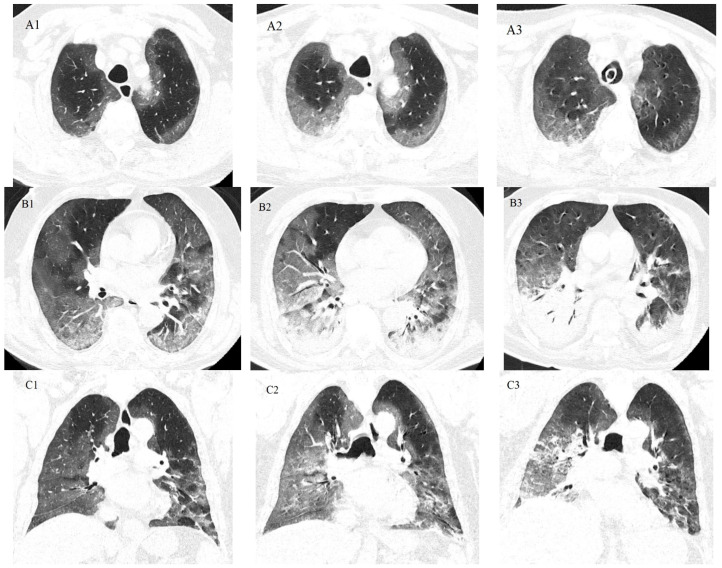
Aspect of unenhanced chest computed tomography (CT) images with evolving changes. Axial CT chest images at the upper lobes level in the fourth (**A1**), ninth (**A2**), and 21st (**A3**) hospitalization days, that suggest progression towards resolution of ground-glass opacities; Axial at lower lobes level and coronal chest CT images in the fourth (**B1**,**C1**), ninth (**B2**,**C2**), and 21st (**B3**,**C3**) hospitalization days, that reveals intensification of pulmonary consolidation, air bronchogram, and pleural effusion moderate on the right, and small on the left.

The first chest X-ray performed on the third day of hospitalization, revealed bilateral ground-glass areas of medium to low radiopacity, located basally and latero-thoracically, with an estimated global pulmonary involvement of 40–45% (Figure 1). During the course of the illness, on the fourth day of hospitalization, the computed tomography (CT) images suggested progression toward resorption (Figure 2(A1–A3,B1,C1)). However, on the ninth day of hospitalization, the CT-imaging showed infrahilar bilaterally, small, well-defined opacities with a tendency to coalesce, occupying approximately 20% of the left lung field and 10–20% of the right lung field (Figure 2(B2,C2)). Globally, the degree of lungs impairment reached 60–70% of their volume and an increasing density has been observed in ground-glass areas. By the 21st day of hospitalization, CT imaging identified areas of consolidation involving almost the entire right lower lobe and the posterior portion of the left lower lobe, presence of air bronchogram, as well as bilateral pleural effusions-moderate on the right and minimal on the left (Figure 2(B3,C3)). The chest X-ray, taken on the 13th day of hospitalization, revealed areas of condensation and bilateral pleural effusions that were similar in description to CT examinations.

Regular bacteriological cultures from blood, tracheal aspirate, and urine initially (on the ninth and 14th days of hospitalization) were negative. Subsequently, a carbapenemase-producing *Klebsiella pneumoniae* strain was identified from blood cultures and tracheal aspirates, both collected on the 19th day of hospitalization. Phenotypic testing (turbidimetric method) showed that the *Klebsiella pneumoniae* strain was resistant to colistin and sensitive to amikacin, as well as to combinations of ceftazidime-avibactam and imipenem-relebactam. Additionally, the same tracheal aspirate sample tested positive for both *Legionella pneumophila* and *Klebsiella pneumoniae* via rt-PCR. Screening conducted on the 15th day of hospitalization identified fecal carriage of vancomycin-resistant *Enterococcus faecium*.

Upon admission, treatment for severe COVID-19 was initiated in accordance with national protocols. This included antiviral therapy with remdesivir (100 mg/day for 5 days) and favipiravir (600 mg every 12 h for 7 days), interleukin-1 receptor antagonist (anakinra 100 mg/day for 7 days), antibiotic (ceftriaxone 2 g/day for 9 days), corticosteroid (dexamethasone 8 mg/day), and anticoagulant therapy (enoxaparin sodium 80 mg twice daily). Oxygen therapy was administered initially via a reservoir mask at 14 L/min, alternating with continuous positive airway pressure (CPAP) with an inspired oxygen fraction (FiO_2_) of 60% and positive end-expiratory pressure (PEEP) of 5 cm H_2_O, and later adjusted according to the level of respiratory failure. Dexamethasone and the anticoagulant were continued throughout the entire hospitalization period.

Antibiotic treatment was continued with meropenem (1 g every 8 h, in prolonged infusion) starting from the ninth day of hospitalization, for 11 days. On the 18th day of hospitalization, colistin (2.5 million IU twice daily) was added and administered until the 22nd day of hospitalization. According to the *Klebsiella pneumoniae* strain’s antibiotic sensitivity, when it became available, ceftazidime-avibactam was added, with only two doses administered before the patient’s death. Treatment for coexisting chronic conditions was maintained throughout the hospitalization, except for the last almost 10 days, during which treatment was focused on managing the septic shock caused by the pulmonary infection.

After admission, during the first three days of hospitalization, the patient’s condition worsened, with deteriorating general status and an oxygen saturation (SpO_2_) measured by pulse oximetry of 91%, under non-invasive oxygen therapy with a reservoir mask. Bilateral crackles were present over 2/3 of the thoracic surface. The patient was then transferred to a tertiary hospital. Further, the patient’s clinical evolution had repeated exacerbation episodes with tachypnea (>35 to 45–50 breaths/min), fever, asthenia, anxiety, psychomotor agitation accompanied by the alteration of general condition. From the fifth day of hospitalization, the cough became productive with muco-purulent sputum (Figure 1). Oxygenation parameters measured by PaO_2_ and Horowitz index (<100 mmHg) despite oxygen therapy remained at low values, indicating in correlation with radiological images, a severe progressive acute respiratory distress syndrome (ARDS) (Table 1, Figure 1). At this stage of the illness’s evolution, the clinical form of COVID-19 has become a critical form. From the 10th day the patient required assistance in intensive care unit, with evolution in progressive worsening. Here, from the 13th day of hospitalization, due to pronounced respiratory failure, the patient was orotracheally intubated and mechanical ventilated under propofol sedation and curarization, and for cardiac failure, vasoactive support with continuous infusion of noradrenaline was administered. On days 15–17 and from day 21, the diuresis had to be stimulated with a loop diuretic (furosemide). The patient’s condition continued to deteriorate, and he passed away on the 23rd day of hospitalization. An autopsy was not performed.

## 3. Discussion

The pulmonary coinfection with *Legionella pneumophila* and *Klebsiella pneumoniae* presented by the patient, according to the case definitions criteria used in European Union, was a probable case of Legionnaire’s disease and a ventilator-associated pneumonia (VAP) with *Klebsiella pneumoniae* [15].

To confirm legionellosis, urinary antigen testing or culture would have been required, but these were not conducted due to the absence of clinical suspicion. However, the method used (rt-PCR) has a specificity exceeding 99% for detecting *L. pneumophila*, differing from urinary antigen testing, which has high specificity only for serogroup 1 of *Legionella pneumophila* [7,16].

Confirming pneumonia caused by *Klebsiella pneumoniae* would have necessitated quantitative assessment and possibly distal sampling through bronchoalveolar lavage or brushing [7,15]. However, criteria for VAP were met through the presence of worsening gas exchange, leukocytosis, CT images, and positive blood culture not related to another source of infection [15]. Post-mortem bacteriological sampling could have supported once again the diagnosis of pulmonary coinfection, but this was not feasible as the bacteriological results were obtained after the patient’s death and his body had already been released to the family.

At the time the suspicion of legionellosis was raised, further environmental sampling for *Legionella pneumophila* could not be performed due to surveillance procedures for legionellosis, which require reporting a legionellosis case to the Public Health Authority [17]. Following that, the authority is the only institution authorized for the collection and management of environmental samples.

In the presented case, the retrospective diagnosis of legionellosis was based on several criteria: worsening symptoms under antibiotic treatment (third-generation cephalosporins and carbapenems), cough with mucopurulent sputum starting from the fifth day of hospitalization, accompanied by fever (38.8 °C), leukocytosis with neutrophilia, negative bacteriological results (from tracheal aspirates and blood cultures), imaging findings of pulmonary consolidation (evidenced by CT on the ninth day) in the posterior regions of the lower lobes, following the progression to resorption of ground-glass opacities (COVID-19 pneumonia), development of pleural effusions, and identification of *Legionella pneumophila* via rt-PCR from tracheal aspirate.

The subsequent onset of pneumonia caused by *Klebsiella pneumoniae* was established based on the imaging changes (CT performed on the 21st day) showing pulmonary consolidation with total involvement of the right lower lobe, pleural effusions, and high colony density (measured semi-quantitatively) of *Klebsiella pneumoniae* in tracheal aspirate culture and blood culture (indicative of bacteremia associated with the pneumonic process). The *Klebsiella pneumoniae* strain, which produced carbapenemase, was resistant even to colistin, explaining the lack of response to the administered antibiotic treatment (carbapenems, colistin) and the progression of infection to sepsis with pulmonary origin and then septic shock. The abundant purulent respiratory secretion in the last two days was due to a tracheitis associated with the tracheal catheter.

In this patient, the only context in which he was exposed to water aerosols was through non-invasive oxygen therapy with a reservoir mask, where the gas mixture was humidified with sterile water heated to 37 °C. Additionally, we cannot exclude a community source since no assessments were conducted either in the hospital or at the patient’s home regarding the contamination level of water sources with *Legionella*, and the incubation period can range from 2 to 14 days or even longer [7]. Imaging findings cannot distinguish between different etiologies or the onset of infection [7,8,9]. In our current practice, microbiological monitoring of *Legionella pneumophila* density in water distributed within hospitals is not performed due to the rarity of diagnosed legionellosis cases. The superinfection with *Klebsiella pneumoniae* was associated with mechanical ventilation, likely resulting from the selection of a resistant strain from the patient’s flora, as a consequence of the antibiotic treatment administered throughout the hospitalization.

The risk for legionellosis is known to be increased (0.4–15% among those with risk factors compared to 0.1–5% among healthy individuals exposed to *Legionella*) in elderly individuals, smokers, those with chronic lung diseases, and those with altered immune responses [7]. In patients with COVID-19, the occurrence of legionellosis may be favored by changes induced by SARS-CoV-2, including lymphopenia, as well as by treatment with corticosteroids (dexamethasone), immunomodulatory treatment, or other possible favorable conditions [14]. The presented patient presented co-morbidities undergoing chronic treatment and colorectal cancer, for which he underwent chemotherapy and radiation treatment, and during hospitalization he was treated for severe COVID-19 with corticosteroids, interleukin-1 receptor antagonist, and antibiotics.

The analysis of this case revealed that in patients with critical COVID-19, the risk of undiagnosed pulmonary coinfections can be attributed to several factors. Firstly, the similarity of symptoms complicates the clinical suspicion of coinfections. Secondly, the infrequent diagnosis of certain pneumonia types in the geographic area, together with the lack of routine confirmation tests for some etiologies in current practice, contributes to the challenge.

To reduce the risk of death in patients within this category, immediate measures may include: advising practitioners to incorporate suspicion of legionellosis into the practical diagnostic guidelines for pneumonias requiring hospitalization, particularly in geographic areas where the condition is rarely diagnosed; acknowledging risk factors to identify patients more likely to develop pulmonary coinfections; use of antibiotics with proven efficacy against legionellosis in the escalation therapy for severe pneumonias pending the establishment of etiology, and ensuring rigorous preparation of medical devices to minimize nosocomial risks. Other measures focus on improving diagnostic capabilities in current practice, by knowing the circulating strains of *Legionella pneumophila* in the geographic area, their antibiotic sensitivity profiles, and by conducting microbiological monitoring of water in healthcare facilities where patients with risk factors for severe legionellosis are treated.

The case novelty consists in the pulmonary concomitant infection with *Legionella pneumophila* and *Klebsiella pneumoniae* in a patient with COVID-19. We have not found any reports of this concomitant association in the literature published so far. Most publications mainly reported the superinfections prevalence in different categories of COVID-19 patient [1,2,3,4,5,6,12]. Furthermore, in non-COVID-19 patients the association of multiple superinfections was reported most in immunocompromised individuals, such as those with organ transplants [9,10]. At the same time, similarly to our observations in the reported case, it is suggested that the SARS-CoV-2 infection can predispose to multiple bacterial superinfections, and the pathological modifications preferentially favor certain infections and associations between pathogens, namely *Legionella pneumophila* and *Klebsiella pneumoniae*, as was the case in our report. These aspects of COVID-19 pathogenesis need to be identified and described through further investigation.

The opportunities provided by this case report included raising awareness on the diagnosis of coinfections, particularly with legionellosis, which are likely more frequent than currently recognized, both as nosocomial and community-acquired pneumonias. Analyzing cases with coinfections can provide medical insights that may enhance diagnostic and therapeutic algorithms for COVID-19. Additionally, identifying risk factors and parameters that can be routinely assessed in laboratories and are correlated with the presence of coinfections would contribute to early diagnosis (or suspicion) and reduce the risk of mortality among hospitalized pneumonia patients, especially in general hospitals within our geographic area.

## 4. Conclusions

The presented case was a pulmonary coinfection with *Legionella pneumophila* and *Klebsiella pneumoniae* in a patient with critical form of COVID-19. Based on the investigation methods available in our current practice, we were able to establish the diagnosis of a probable case of Legionnaire’s disease and a VAP with *Klebsiella pneumoniae*. 

## Figures and Tables

**Table 1:** Laboratory investigations performed during patient’s hospitalization. Only the parameters that have had changes compared to the reference values are presented (these and the unit of measure are specified in parenthesis). The reference ranges for the parameters are the ones provided by the laboratory.

Laboratory Parameter	Hospital Day														
	1	4	5	7	8	9	10	12	13	15	16	17	18	21	23
pO_2_^1^ (83–108 mmHg ^8^)	-	-	-	82	71	57	62	57	65	63	56	53	71	51	47
AST ^2^ (0–45 U/L ^9^)	55	105	68	28	40	30	112	149	63	183	65	50	230	88	46
ALT ^3^ (0–45 U/L)	37	183	152	71	65	61	125	215	178	297	193	140	323	363	206
LDH ^4^ (0–250 U/L)	-	421	433	-	441	-	471	-	-	360	-	307	-	414	-
Urea (0–50 mg/dL ^10^)	38.40	48	-	50	54	68	82	79	76	62	82	97	108	119	102
K ^5^ (3.5–5.1 mEq/L ^11^)	2.8	2.9	3.5	-	3.6	-	-	-	-	-	-	-	-	-	-
ESR ^6^ 1 h (<20 mm ^12^)	30	44	-	-	-	38	-	-	-	-	-	-	-	-	-
Fibrinogen (180–400 mg/dL)	462	629.14	586	-	610.53	-	730.01	730.01	-	-	924.86	-	-	-	-
Ferritin (23.9–336.2 ng/mL ^13^)	-	873.50	-	568.20	674.80	-	-	-	-	-	-	4 023	-	-	-
RBCs ^7^ (4.32–5.66 × 10^6^/µL ^14^)	4.44	4.31	4.57	4.09	4.04	4.18	4.15	4.23	3.64	3.57	3.67	3.90	4.07	3.70	3.30
Hemoglobin (13.3–17.6 g/dL ^15^)	14.80	14.40	14.80	13.70	13.40	13.80	13.60	14	11.90	11.90	12	12.80	13.30	12.10	10.70
Hematocrit (39.0–51.0%)	42.20	42	42.20	39.80	37.80	39.20	39.20	40.10	36.10	35.40	35.90	38.50	40.90	36.80	32.80

^1^ pO_2_: arterial partial pressure of oxygen; ^2^ AST: aspartate aminotransferase; ^3^ ALT: alanine aminotransferase; ^4^ LDH: lactic dehydrogenase; ^5^ K: potassium; ^6^ ESR: erythrocyte sedimentation rate; ^7^ RBCs: red blood cells; ^8^ mmHg: millimeters mercury column; ^9^ U/L: units to liter; ^10^ mg/dL: milligrams to deciliter; ^11^ mEq/L: milliequivalents to liter; ^12^ mm: millimeter; ^13^ ng/mL nanograms to milliliters; ^14^ µL: microliter; ^15^ g/dL: gram per deciliter.

## Data Availability

Data supporting the study results can be provided followed by reasonable request sent to the corresponding author’s e-mail.

## References

[1] Zamora-Cintas M.I., López D.J., Blanco A.C., Rodriguez T.M., Segarra J.M., Novales J.M., Ferriol M.F.R., Maestre M.M., Sacristán M.S. (2021). Coinfections among hospitalized patients with covid-19 in the first pandemic wave. Diagn. Microbiol. Infect. Dis..

[2] Hughes S., Troise O., Donaldson H., Mughal N., Moore L.S.P. (2020). Bacterial and fungal coinfection among hospitalized patients with COVID-19: A retrospective cohort study in a UK secondary-care setting. Clin. Microbiol. Infect..

[3] Husain M., Valayer S., Poey N., Rondinaud E., d’Humières C., Visseaux B., Lariven S., Lescure F.X., Deconinck L. (2022). Pulmonary bacterial infections in adult patients hospitalized for COVID-19 in standard wards. Infect. Dis. Now..

[4] Rothe K., Feihl S., Schneider J., Wallnöfer F., Wurst M., Lukas M., Treiber M., Lahmer T., Heim M., Dommasch M. (2021). Rates of bacterial co-infections and antimicrobial use in COVID-19 patients: A retrospective cohort study in light of antibiotic stewardship. Eur. J. Clin. Microbiol. Infect. Dis..

[5] Lansbury L., Lim B., Baskaran V., Lim W.S. (2020). Co-infections in people with COVID-19: A systematic review and meta-analysis. J. Infect..

[6] Fattorini L., Creti R., Palma C., Pantosti A., Unit of Antibiotic Resistance and Special Pathogens (2020). Bacterial coinfections in COVID-19: An underestimated adversary. Ann. Ist. Super. Sanita.

[7] Yu V.L., Pedro-Botet M., Lin Y.E., Jameson J., Fauci A.S., Kasper D.L., Hauser S.L., Longo D.L., Loscalzo J. (2018). Legionella infections. Harrison’s Principles of Internal Medicine, 20e.

[8] Center for Disease Control and Prevention (2024). *Legionella* (Legionaires’ Disease and Pontiac Fever). About Legionnaires’ Disease. https://www.cdc.gov/legionella/about/index.html.

[9] Scaturro M., Girolamini L., Pascale M.R., Mazzotta M., Marino F., Errico G., Monaco M., Girolamo A., Rota M.C., Ricci M.L. (2022). Case Report: First Report of Fatal *Legionella pneumophila* and *Klebsiella pneumoniae* Coinfection in a Kidney Transplant Recipient. Front. Med..

[10] Takayanagi N., Tokunaga D., Matsushima H., Ubukata M., Sato N., Kurashima K., Yanagisawa T., Sugita Y., Kanazawa M. (2004). Polymicrobial infections in patients with Legionella pneumonia. Nihon Kokyuki Gakkai Zasshi.

[11] National Center for Surveillance and Control of Communicable Diseases—National Institute of Public Health Analysis of Cases of Pneumonia with *Legionella pneumophila*. https://insp.gov.ro/centrul-national-de-supraveghere-si-control-al-bolilor-transmisibile-cnscbt/analiza-date-supraveghere/.

[12] Allam C., Gaymard A., Descours G., Ginevra C., Josset L., Bouscambert M., Beraud L., Ibranosyan M., Golfier C., Friggeri A. (2021). Co-infection with Legionella and SARS-CoV-2, France, March 2020. Emerg. Infect Dis..

[13] Fareau G.G., Vassilopoulou-Sellin R. (2007). Hypercortisolemia and infection. Infect. Dis. Clin. N. Am..

[14] Shapiro L., Scherger S., Franco-Paredes C., Gharamti A., Henao-Martinez A.F. (2023). Anakinra authorized to treat severe coronavirus disease 2019; Sepsis breakthrough or time to reflect?. Front. Microbiol..

[15] Commission Implementing Decision (EU) 2018/945 of 22 June 2018 on the Communicable Diseases and Related Special Health Issues to be Covered by Epidemiological Surveillance as Well as Relevant Case Definitions. https://eur-lex.europa.eu/eli/dec_impl/2018/945/oj.

[16] Center for Disease Control and Prevention (2024). *Legionella* (Legionaires’ Disease and Pontiac Fever). Laboratory Testing for *Legionella*. https://www.cdc.gov/legionella/php/laboratories/index.html.

[17] National Center for Surveillance and Control of Communicable Diseases—National Institute of Public Health Pneumonia with Legionella. Surveillance Methodology. https://insp.gov.ro/centrul-national-de-supraveghere-si-control-al-bolilor-transmisibile-cnscbt/metodologii/.

